# Fibrinolytic suppression and increased uterine artery resistance are associated with embryo resorption in DBA/2 mated CBA/J mice

**DOI:** 10.3389/fphys.2026.1942168

**Published:** 2026-10-12

**Authors:** Youchen Liu, Jingjing Wang, Boning Huang, Ping Tang, Fangle Liu, Yuying Zheng, Yilin Li, Huiyi Xie, Liuqing Lin, Xinrong Yang, Xiubing Zhang, Jie Gao, Baoqin Lin

**Affiliations:** The First Affiliated Hospital of Guangzhou University of Chinese Medicine, Guangdong Clinical Research Academy of Chinese Medicine, Guangdong Engineering Research Center of Commercialization of Medical Institution Preparations and Traditional Chinese Medicines, Guangdong Engineering Technology Research Center of Commercialization of Lingnan Special Medical Institution Preparations, Guangzhou, Guangdong, China

**Keywords:** DBA/2 mated CBA/J, fibrinolytic suppression, prothrombotic state, RPL, uterine artery resistance

## Abstract

**Objective:**

This study aimed to characterize changes in prothrombotic state (hypercoagulability, hypofibrinolysis, and hemodynamic dysfunction) in DBA/2 mated CBA/J mice from embryonic day (E) 9.5 to E13.5 and to identify factors independently associated with embryo resorption and placental congestion.

**Method:**

CBA/J females were mated with DBA/2 and BALB/c males to establish the abortion-prone model and normal control, respectively. Pregnant mice were divided into normal, model, dydrogesterone (DYD), and enoxaparin sodium (ENOX) groups. Six mice per group were sacrificed at E9.5, 11.5, and 13.5. Hemodynamic parameters were assessed by Doppler ultrasound, and coagulation and fibrinolysis markers were measured in serum and placental tissues. Moreover, the univariate and multivariate regression analyses were performed to identify independently associated factors of embryo resorption and placental congestion.

**Results:**

From E9.5 to E13.5, DBA/2 mated CBA/J mice exhibited progressive hypercoagulability and fibrinolytic inhibition, with embryo resorption peaking at E13.5. On E13.5, model mice showed elevated uterine and umbilical artery resistance, decreased serum fibrinogen (FIB) and tissue-type plasminogen activator (t-PA), and elevated D-dimer and plasminogen activator inhibitor-1 (PAI-1). The placental congestion, fibrin thrombi, and collagen deposition were observed in model mice, alongside increased expression of FIB, tissue factor, and PAI-1, and reduced t-PA. DYD ameliorated embryo resorption, decreased arterial resistance and placental PAI-1. ENOX also decreased arterial resistance, but failed to rescue embryo resorption. Furthermore, multivariate regression identified uterine artery PI and RI, along with serum t-PA and PAI-1 as independently associated factors of embryo resorption, whereas uterine artery PI and RI, along with t-PA and APTT associated with placental congestion.

**Conclusion:**

DBA/2 mated CBA/J mice exhibit key hypercoagulable and hypofibrinolytic features of RPL with prothrombotic state. Uterine artery PI and RI, serum t-PA and PAI-1 may serve as potential indicators for embryo resorption and placental congestion.

## 1 Introduction

Recurrent pregnancy loss (RPL), defined as two or more pregnancy losses before 28 weeks after comprehensive evaluation, may affect 10% to 50% of women aged 20 to 45 years. (Practice Committee of the American Society for Reproductive Medicine, 2020; Obstetrics Subgroup, Chinese Society of Obstetrics and Gynecology, 2022). This condition represents one of the major challenges in reproductive medicine, particularly in the context of delayed childbearing and declining fertility rates (Bender Atik et al, 2023).

Clinically, RPL patients with a prothrombotic state, characterized by hypercoagulability and hypofibrinolysis, are at a higher risk of pregnancy complications (Cho et al., 2021; Di Micco, 2008; Yagmur et al., 2019). Activated partial thromboplastin time (APTT) and prothrombin time (PT) do not differ significantly (Ibrahim et al., 2025; Samfireag et al., 2023), whereas fibrinogen (FIB) level decreases and D-dimer (DD) rises, accompanied by elevated plasminogen activator inhibitor-1 (PAI-1) (Maghsudlu et al., 2024; Ye et al., 2022), though the results vary across studies. In addition, hemodynamic dysfunction has been observed in RPL, manifesting as elevated uterine artery resistance (Pang et al., 2025). Nevertheless, the association of these parameters with pregnancy loss remains unclear.

The DBA/2 mated CBA/J mouse model is widely recognized as a classic model of RPL (Shi et al., 2025; Zarnani et al., 2024). Coagulation abnormalities have been investigated in this model, including upregulated decidual tissue factor (TF) and fibrin deposition (Clark et al., 1999; Girardi et al., 2006). However, whether this model exhibits a prothrombotic state (hypercoagulability, hypofibrinolysis, and hemodynamic dysfunction) remains unknown. In addition, the association between these prothrombotic state parameters and pregnancy loss has not been established.

Notably, embryonic day (E) 9.5 to E13.5 is both the stage with a significant increase in embryo resorption rate and a critical period for placental vascular development and remodeling (Croy et al., 2018; Giugliano et al., 2025). During this period, the coagulation-fibrinolysis abnormalities and hemodynamic changes may interact to constitute the pathological basis of microthrombus formation and placental congestion. Nevertheless, previous studies have predominantly adopted single-timepoint designs without systematically tracking the dynamic changes of these parameters within this window (Clark et al., 1999; Lin et al., 2011; Redecha et al., 2009).

Hence, in this study, we focused on identifying the changes in coagulation, fibrinolysis, and hemodynamics in DBA/2 mated CBA/J mice from E9.5 to E13.5. Additionally, the univariate and multivariate regression analyses were performed to evaluate the associations of Doppler indices and serum markers with embryo resorption and placental congestion. These findings may provide a basis for mechanism studies of RPL with prothrombotic state and offer a preclinical model for pharmacodynamic and risk evaluation in drug development.

## 2 Materials and methods

### 2.1 Animal

Eighteen BALB/c male mice (9–10 weeks old), fifty-four DBA/2 male mice (9–10 weeks old) and seventy-two CBA/J female mice (8–9 weeks old) were purchased from Beijing HFK Bioscience Co., Ltd (Approved No. SCXK (Jing) 2024-0003, Beijing, China). All mice were housed in the Experimental Center of the First Affiliated Hospital of Guangzhou University of Traditional Chinese Medicine (SYXK (Yue) 2023-0092). Mice had free access to a standard diet and water in a room maintained at 24 ± 1°C with a 12 h/12 h cyclic lighting schedule. All experiments were approved by the Animal Care Welfare Committee of the First Affiliated Hospital of Guangzhou University of Traditional Chinese Medicine (Approved No. GZTCMF1-20240106), with corresponding animal experiment certificate (No.00449851).

### 2.2 Animal treatments

Females and males were mated at a 1:1 ratio overnight (Zarnani et al., 2024). Pregnant mice were identified by vaginal plug visualization the next morning. If a vaginal plug was detected, this day was recognized as the embryonic day 0.5 (E0.5) (Yang et al., 2023). At E0.5, the pregnant CBA/J females mated with BALB/c males were classified as the normal group (*n* = 18). The pregnant CBA/J females mated with DBA/2 males were randomly divided into 3 groups (*n* = 18 per group): the model group, DYD group (preferred oral progestogen for RPL), and ENOX group (the standard anticoagulant during gestation). Six mice in each time points (E9.5, E11.5 and E13.5) were set up in each group.

As shown in **Figure 1A**, the mice in both normal and model groups were received with distilled water (10 mL/kg) by oral gavage from E0.5 until the designated day. Mice in the DYD group received daily oral gavage of DYD (3 mg/kg/d; HJ20170221, Abbott Biologicals B.V., Netherlands). Mice in the ENOX group received daily subcutaneous injection of ENOX (600 anti-Xa IU/kg/d; J20180036, Sanofi Winthrop Industrie, France) dissolved in 5% glucose solution (Infectiology Group, Chinese Society of Hematology, Chinese Medical Association et al., 2025). Both treatments were initiated at E0.5 and continued until the designated day.

**Figure 1:**
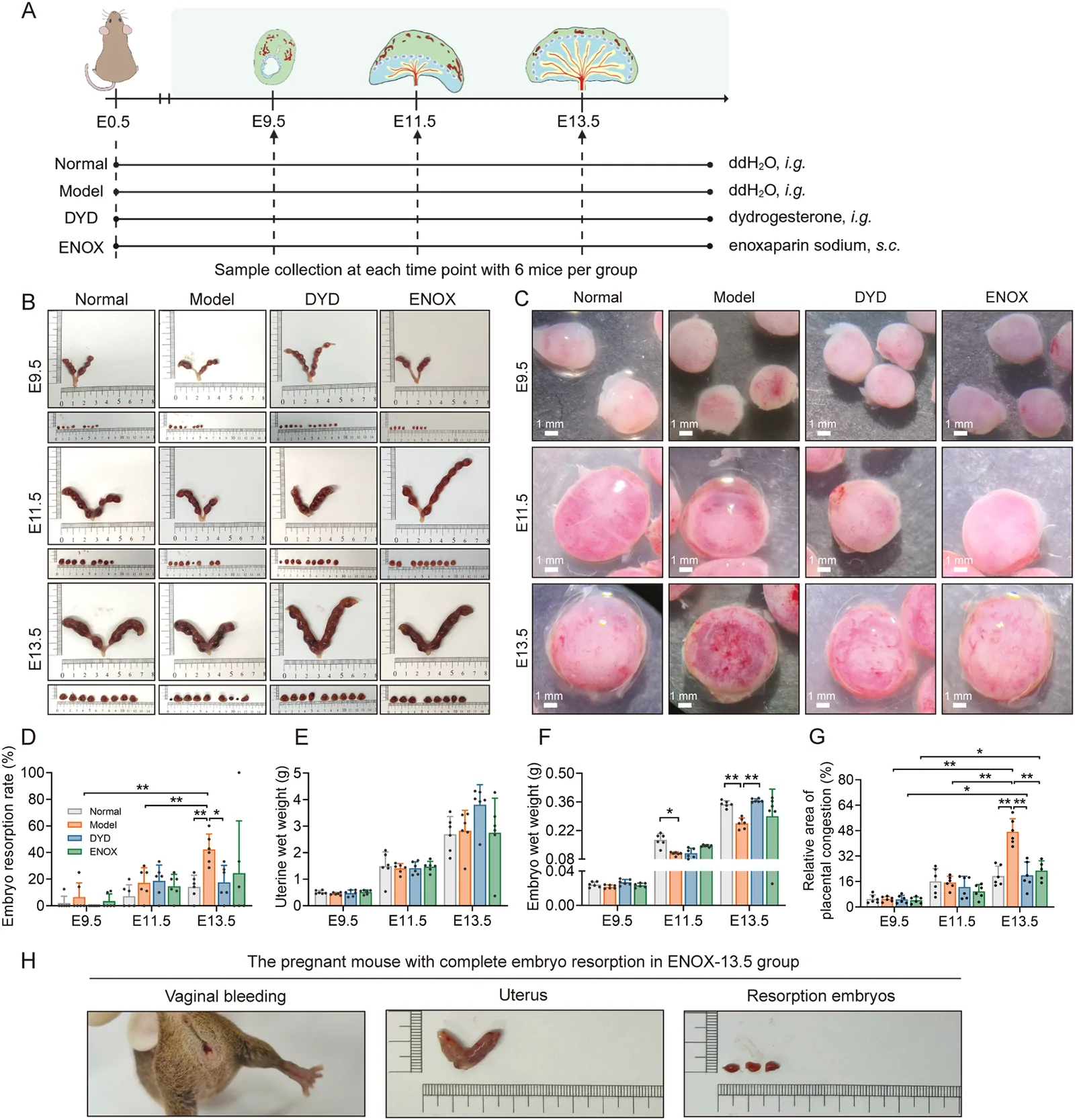
Pregnancy progression in DBA/2 mated CBA/J mice and BABL/C mated CBA/J mice at different embryonic days. **(A)** The diagram of drugs administration. **(B)** Representative morphology images of the uteri and embryos. **(C)** Representative images of placenta under a stereomicroscope. **(D)** Embryo resorption rate. **(E)** Uterine wet weight. **(F)** Embryo wet weight. **(G)** The relative area of placental congestion. **(H)** Vaginal bleeding with complete embryonic loss in the ENOX group at E13.5. Data were expressed as mean ± SD, *n* = 6 per group other than *n* = 5 for E13.5-ENOX group. ^*^P < 0.05 and ^**^P < 0.01.

### 2.3 Doppler ultrasound

At E9.5, E11.5 and E13.5, the ultrasound examinations of pregnant mice were performed using the M90 SCI system (Shenzhen Mindray Animal Medical Technology Co., Ltd., Shenzhen, China). Briefly, mice were anesthetized with isoflurane. The abdominal fur was removed by shaving. Subsequently, mice were placed on a thermostatically controlled heating pad to maintain body temperature. Using acoustic coupling gel, the transducer was placed gently on the shaved abdomen. A L20-5S ultrasonic probe was employed to obtain velocity waveforms from the uterine arteries supplying the implantation sites. Peak systolic velocity (PSV), end diastolic velocity (EDV) and mean velocity (MV) were recorded (Tan et al., 2024). Three consecutive pulse waves were calculated and the average was considered as one trial. Resistance index (RI), pulsatility index (PI) and systolic/diastolic ratio (S/D) were calculated as follows: RI = (PSV-EDV)/PSV, PI = (PSV-EDV)/MV, and S/D = PSV/EDV.

### 2.4 Sample collection

Following ultrasound examination at designated time points (E9.5, E11.5, E13.5), peripheral blood was collected from mice under anesthesia, and the mice were sacrificed to harvest uteroplacental units. The wet weights of uterine and fetal were measured. Blood anticoagulated with sodium citrate was centrifuged at 3000 rpm for 10 min to obtain plasma for the detection of APTT and PT. Non-anticoagulated blood was kept at room temperature for 2 h, and centrifugated at 3000 rpm for 15 min to obtain serum for Enzyme linked immunosorbent assay (ELISA).

### 2.5 Assessment of embryonic resorption rate and relative area of placental congestion

Embryo absorption rates were calculated as follows:


$$Embryos\;resorption\;rate\;\left(\%\right)=\frac{\text{Reabsorbed embryos}}{\text{Viable embryos}+\text{Reabsorbed embryos}}\times 100\%$$

Placental maternal-side blood stasis area was quantified utilizing Image J software (National Institutes of Health, Maryland, USA). The relative area of placental congestion was calculated as follows (Ming et al., 2021):


$$Relative\;area\;of\;placental\;congestion\;\left(\%\right)=\frac{Placental\;congestion\;area}{Total\;placental\;area}\times 100\%$$

### 2.6 Histological analysis

Briefly, the whole embryos were fixed in 4% paraformaldehyde for 48 h and then dehydrated by gradient ethanol, xylene transparency, and embedded in paraffin (Chen et al., 2024). Transverse sections (4 μm) through the largest placental cross-section containing the intact fetoplacental interface were mounted. Tissue sections were sequentially stained with hematoxylin and eosin (HE; DH0006, Beijing Leagene Biotechnology Co., Ltd., Beijing, China) for morphology, and Masson’s trichrome (G1346, Beijing Solarbio Science & Technology Co., Ltd., Beijing, China) to quantify collagen deposition. The images were acquired using a digital slide scanner (DX150; 3DHISTECH Ltd., Budapest, Magyarország).

For morphological analysis, at E9.5, arteries were identified and counted in six random fields per group at 50× and 400× magnification (Pijnenborg, 2015). At E11.5 and E13.5, six random fields per group per time point were examined at 50×, 200×, and 400× magnification. Placental regions were outlined by freehand contour tracing in CaseViewer software (version 2.4; 3DHISTECH Ltd., Budapest, Magyarország), thrombi were then identified and regional thickness was measured (Hosseini et al., 2020). Maternal labyrinthine capillaries were classified as fetal (nucleated red blood cells) or maternal (enucleated red blood cells) and the latter were counted (Pijnenborg, 2015). Only capillaries with a clear, closed lumen and intact endothelium were included. Collapsed or oblique sections were excluded. Manual counting was performed at 400× magnification, with each field of view covering a fixed area of 480 μm × 281 μm. For each animal, six random fields were examined and averaged to obtain a single representative value. All counts and thickness data were expressed as fold changes relative to the corresponding normal group at each time point. All assessments were undertaken by two independent investigators blinded to the group.

For collagen quantification, six random fields at 400× magnification in Masson-stained sections were analyzed using ImageJ software to record collagen-positive area and integrated optical density (IOD). Data were presented as the ratio of collagen-positive to total tissue area, expressed as fold changes relative to the normal group.

### 2.7 Immunohistochemistry assay

After dewaxing and hydration, 4 μm sections were treated with 3% hydrogen peroxide for 20 min to block endogenous peroxidase. Antigen retrieval was performed in EDTA-Na_2_ solution (pH 8.0) for 15 min. Sections were blocked with 10% goat serum at 37°C for 30 min, and then incubated with primary antibodies of anti-fibrinogen (anti-FIB; 1:2000, ab3429, Abcam, Cambridge, MA) or anti-TF (1:200, ET1611-95, Hangzhou huaan biotechnology Co., Ltd., Hangzhou, China) at 4°C overnight. After phosphate-buffered saline (PBS) washing, sections were incubated with HRP-conjugated goat anti-rabbit IgG (1:200, Cell Signaling Technology, Danvers, MA) at room temperature for 1 h. Following PBS washing, 3, 3’-diaminobenzidine staining (Zhongshan Goldenbridge Biotechnology Co., Ltd., Beijing, China) was performed, followed by hematoxylin counterstaining, dehydration and mounting. The images were acquired using a digital slide scanner (DX150; 3DHISTECH Ltd., Budapest, Magyarország). Six randomly selected fields at 400× magnification were analyzed using ImageJ software. Protein expression levels, quantified by the mean IOD/area ratio, were presented as fold changes relative to the normal group.

### 2.8 Assessment of coagulation and fibrinolysis parameters

APTT and PT was detected using assay kits (TC0306 and TC0307, Beijing Leagene Biotechnology Co., Ltd., Beijing, China). The serum level of DD (SYP-M0121) was tested using an ELISA kit (Youpin Biological Technology Co., Ltd., Shenzhen, China). Serum level of FIB (RX202552M), as well as serum and placenta levels of plasminogen activator inhibitor-1 (PAI-1, RX202553M) and tissue-type plasminogen activator (t-PA, RX202381M), were determined using ELISA kits (Quanzhou Ruixin Biotechnology Co., Ltd., Quanzhou, China).

### 2.9 Statistics analysis

Statistical analysis was performed using SPSS software (version 20; IBM, Armonk, NY). Figure plotting was performed using GraphPad Prism (version 10; GraphPad Software Inc, La Jolla, CA). The normality (Shapiro-Wilk test) and homogeneity of variances (F-Test) of data were examined. Data were expressed as the mean ± standard deviation (SD). A *P*-value less than 0.05 was considered statistical significance.

For group comparisons, statistical differences among different groups were analyzed using one-way ANOVA. The Bonferroni test was used for data with equal variance, while Dunnett’s T3 test was applied to analyze data with unequal variance.

For association and predictive analyses, correlations were assessed using Spearman’s rank coefficient. Candidate factors with *P* < 0.20 in univariate linear regression were entered into multivariable linear regression using the enter method for the primary analysis (*n* = 71). Multicollinearity was diagnosed using the variance inflation factor (VIF), with a threshold of less than 5. Hierarchical regression evaluated the incremental value of umbilical artery parameters in the 46-case subset (with both models based on the same subset due to listwise deletion), using ΔR² with F-test P value to assess incremental contribution (Hosmer et al., 2013).

## 3 Results

### 3.1 The placental congestion and ENOX-induced vaginal bleeding in DBA/2 mated CBA/J mice at E13.5

From E9.5 to E13.5, embryos in the normal group developed progressively and exhibited a characteristic string-of-beads arrangement, whereas those in the model group appeared unevenly distributed, puny, and congested (**Figures 1B, C**). In the model group, the embryo resorption rate (**Figure 1D**) and placental congestion area (**Figure 1G**) increased progressively, with the resorption rate at E13.5 increasing by 5.48-fold compared to that at E9.5 (*P* < 0.05) and by 1.46-fold compared to that at E11.5 (*P* < 0.01).

At E13.5, compared to the normal group, the model group exhibited a 2-fold increase in resorption rate (*P* < 0.01, **Figure 1D**), a 25.71% reduction in embryo wet weight (*P* < 0.01, **Figure 1F**) and a 1.43-fold increase in placental congestion area (*P* < 0.01, **Figure 1G**). Compared to the model group, DYD reduced embryo resorption rate by 58.56% (*P* < 0.05), increased embryo wet weight by 42.31% (*P* < 0.01) and reduced placental congestion area by 57.84% (*P* < 0.01). In contrast, ENOX treatment decreased placental congestion area by 51.58% (*P* < 0.01), but had no effect on resorption rate or embryo wet weight. Notably, one ENOX-treated mouse exhibited vaginal bleeding accompanied by complete embryonic resorption at E13.5 (**Figure 1H**).

### 3.2 The progressive elevation of uterine and umbilical arterial resistance in the DBA/2 mated CBA/J mice from E9.5 to E13.5

The hemodynamic characteristics of the uterine and umbilical arteries were assessed by PI, RI and S/D ratio (Velauthar et al., 2014; Skow et al., 2019). From E9.5 to E13.5, uterine artery PI, RI, and S/D remained stable in the normal group, but increased progressively in the model group (**Figures 2A–D**). At E13.5, compared to the normal group, the model group exhibited 66.03%, 42.74% and 21.06% (*P* < 0.05 or *P* < 0.01) increases in uterine artery PI, RI and S/D ratio, respectively. Compared to the model group, DYD reduced PI, RI and S/D by 45.16%, 32.40% and 25.83% (*P* < 0.05 or *P* < 0.01), respectively. Similarly, ENOX treatment reduced PI, RI, and S/D by 37.63%, 27.60% and 20.66% (all *P* < 0.05), respectively.

**Figure 2:**
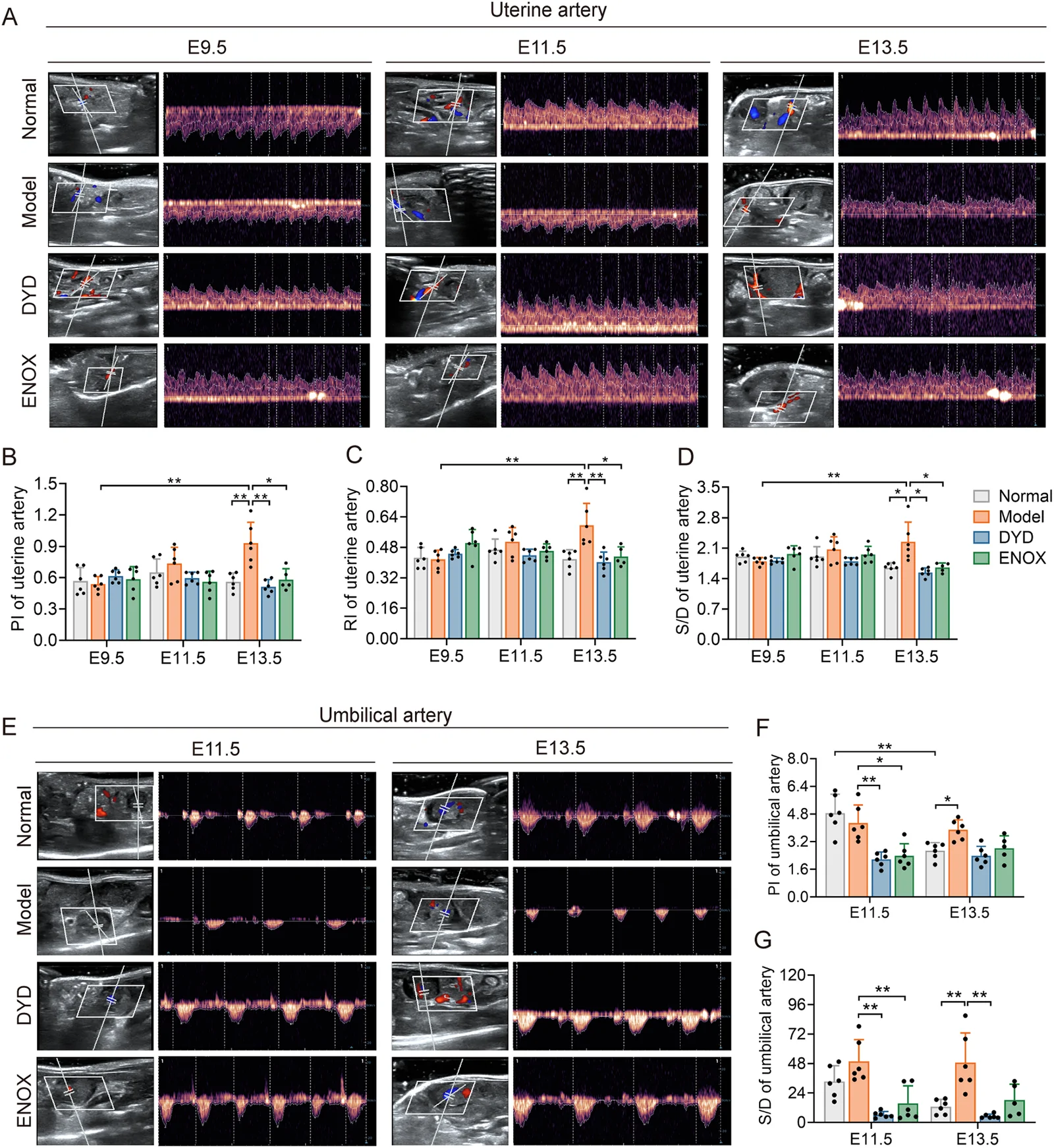
Uterine and umbilical artery hemodynamics in DBA/2 mated CBA/J mice and BABL/C mated CBA/J mice at different embryonic days. **(A)** Doppler ultrasound of uterine artery flow. **(B)** The PI of uterine artery. **(C)** The RI of uterine artery. **(D)** The S/D of uterine artery. **(E)** Doppler ultrasound of umbilical artery flow. **(F)** The PI of umbilical artery. **(G)** The S/D of umbilical artery. Data were expressed as mean ± SD, *n* = 6 per group other than *n* = 5 for E13.5-ENOX group. ^*^P < 0.05 and ^**^P < 0.01.

The umbilical artery RI was not evaluated at E9.5 due to incomplete vascular development (Pijnenborg, 2015). From E11.5 to E13.5, the umbilical artery PI decreased progressively in the normal group (*P* < 0.01, **Figure 2F**), whereas the model, DYD, and ENOX groups showed no significant changes. At E13.5, the model group exhibited a 45.54% increase in PI (*P* < 0.05) and a 2.79-fold elevation in S/D ratio (*P* < 0.01) compared to the normal group. DYD treatments significantly reduced S/D ratios by 89.45% (*P* < 0.01).

### 3.3 The progressive decidual and labyrinthine histopathological deterioration in DBA/2 mated CBA/J mice from E9.5 to E13.5

At E9.5, the model group exhibited a 38.16% reduction in the number of decidual arterial vessels (*P* < 0.05 *vs*. normal group, **Figures 3A, B**). At E11.5, the placental labyrinth thickness in the model group decreased by 47.56% (*P* < 0.05 *vs*. normal group, **Figures 3C, F**), with vascular red blood cells accumulation and occasional fibrin thrombi. At E13.5, the model group showed reductions of 31.41% in junctional thickness, 36.26% in labyrinth thickness, and 35.65% in maternal labyrinth capillaries (*P* < 0.05 *vs*. normal group). Additionally, morphological abnormalities included obscured junctional boundaries, extensive erythrocyte accumulation in decidual sinuses and spiral arteries, dense fibrin thrombi, reduced labyrinth vascularity, and spiral artery stenosis with wall thickening (**Figures 3E–G**; **Supplementary Figure S1**).

**Figure 3:**
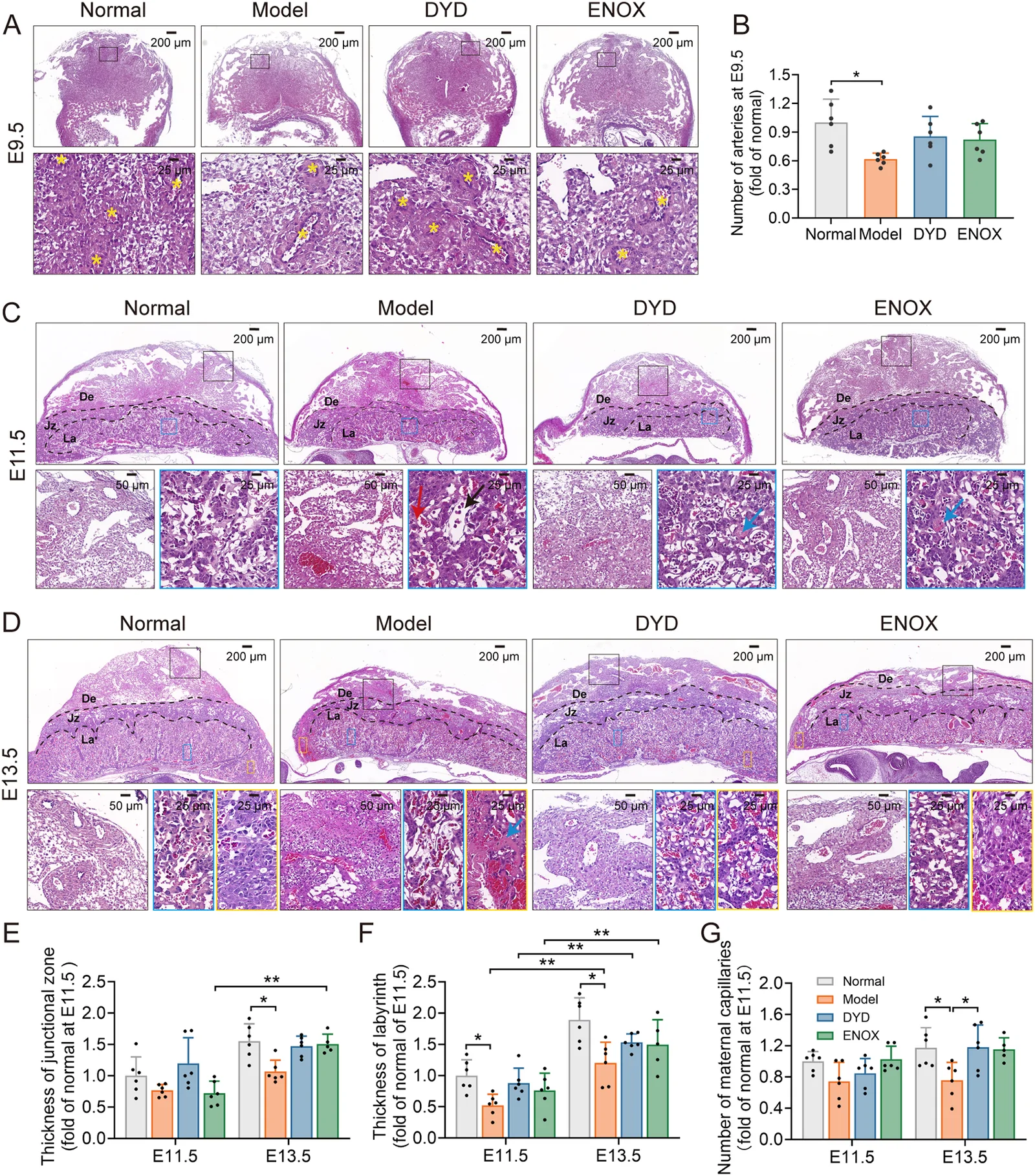
The changes on histopathology of placenta in DBA/2 mated CBA/J mice and BABL/C mated CBA/J mice at different embryonic days. **(A)** Representative images of placental histology at E9.5. **(B)** Number of arteries at E9.5. **(C)** Representative images of placental histology at E11.5. **(D)** Representative images of placental histology at E13.5. The yellow stars represented the arteries in the decidua area, the red arrows pointed to the maternal capillaries, the black arrows pointed to the fetal capillaries, and the blue arrows pointed to the fibrin thrombi. **(E)** Thickness of junctional zone at E11.5 and E13.5. **(F)** Thickness of labyrinth at E11.5 and E13.5. **(G)** Number of maternal capillaries at E11.5 and E13.5. Data were expressed as mean ± SD, n=6 per group other than n=5 for E13.5-ENOX group. *P < 0.05 and **P < 0.01. De, decidua; Jz, junctional zone; La, labyrinth.

Compared to the model group, DYD showed a non-significant increasing trend in junctional zone thickness at E11.5, but significantly increased maternal capillaries at E13.5 (*P* < 0.05). However, ENOX showed no improvement on decidual and labyrinths parameters from E9.5 to E13.5 (**Figures 3B, E–G**).

### 3.4 The coagulation activation and fibrinolysis suppression in the circulatory system of DBA/2 mated CBA/J mice

APTT and PT did not change in normal or model group from E9.5 to E13.5 (**Figures 4A, B**). In this period, the normal group exhibited progressively decreased serum D-D and t-PA levels (*P* < 0.01, **Figures 4D, E**), while FIB and PAI-1 remained unchanged. In contrast, the model group showed no changes in D-D and FIB levels but displayed progressively decreased t-PA and increased PAI-1 (*P* < 0.01, **Figures 4E, F**).

**Figure 4:**
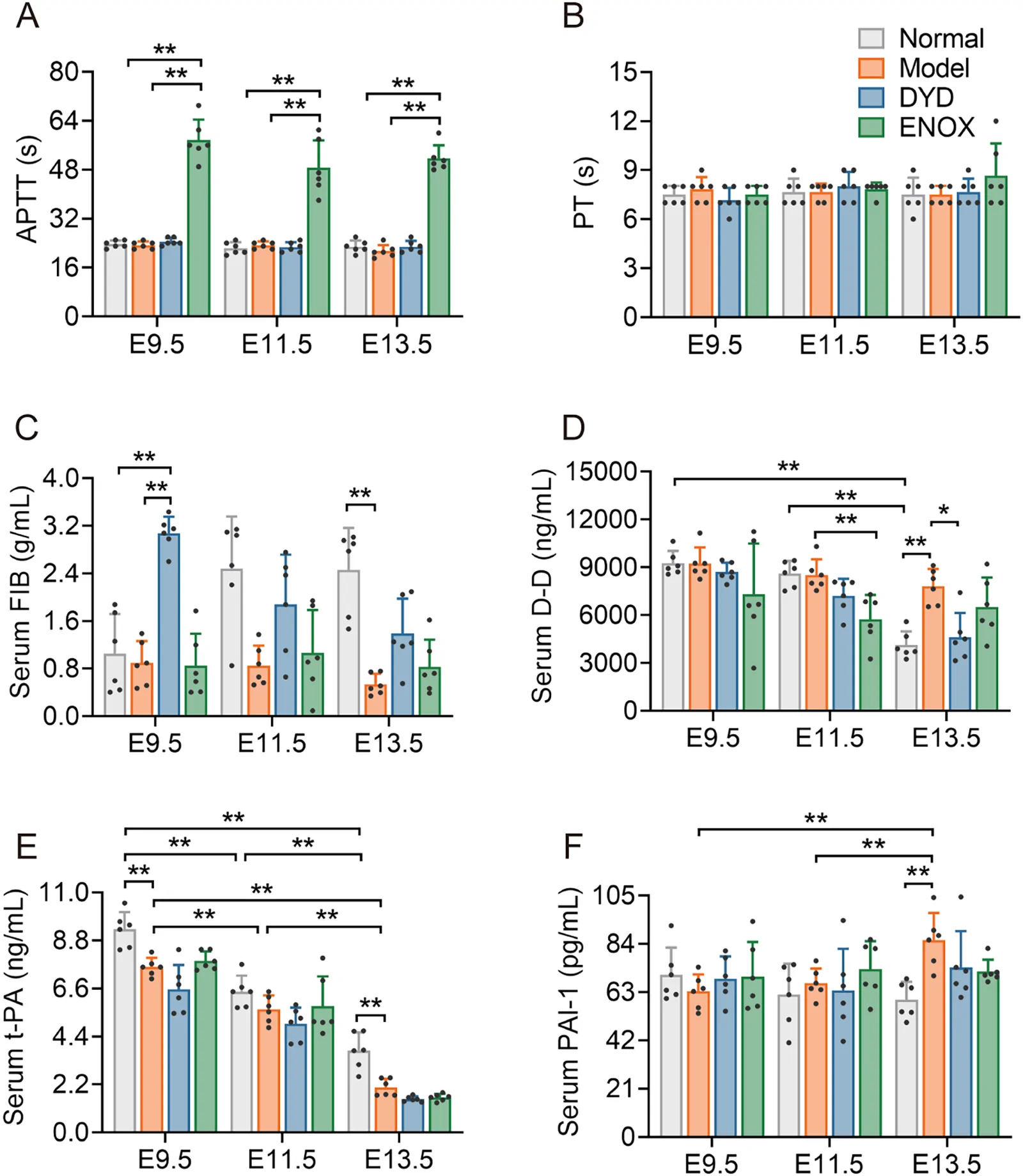
The changes on systemic circulatory coagulation and fibrinolytic function in DBA/2 mated CBA/J mice and BABL/C mated CBA/J mice at different embryonic days. **(A)** APTT. **(B)** PT. **(C)** FIB level in serum. **(D)** D-D level in serum. **(E)** t-PA level in serum. **(F)** PAI-1 level in serum. Data were expressed as mean ± SD, *n* = 6. ^*^P < 0.05 and ^**^P < 0.01.

At E13.5, compared to the normal group, the model group showed a 78.15% decrease in the serum FIB, an 89.23% increase in the serum D-D, a 44.96% reduction in the serum t-PA and a 43.44% increase in the serum PAI-1 (all *P* < 0.01, **Figures 4C–F**). Compared to the model group, DYD increased the serum FIB 2.44-fold at E9.5 (*P* < 0.01) and decreased the serum D-D by 40.97% at E13.5 (*P* < 0.05, **Figures 4C, D**). ENOX prolonged APTT 1.26-fold compared to the normal group (*P* < 0.01) and 1.42-fold compared to the model group (both *P* < 0.01), but did not affect other parameters.

### 3.5 The local coagulation disorders in the placenta of DBA/2 mated CBA/J mice

From E9.5 to E13.5, the model group showed decreased FIB protein expression in the decidua (*P* < 0.01, **Figures 5A, B**) but increased FIB protein expression in the labyrinth (*P* < 0.01, **Figures 5A, C**), with concurrent increased TF protein expression in both the decidua and labyrinth (both *P* < 0.01, **Figures 5D–F**).

**Figure 5:**
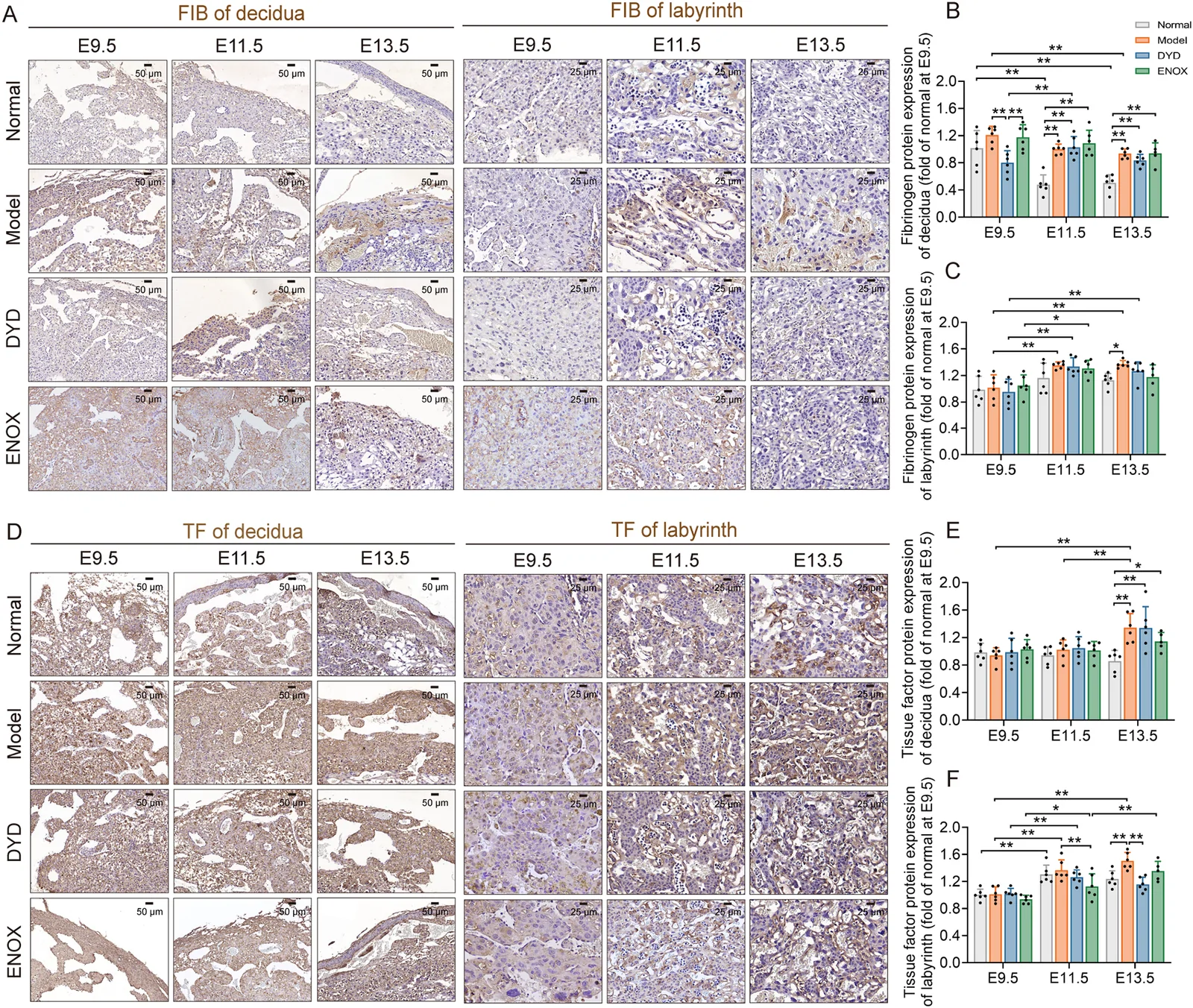
The changes on placental coagulation function in DBA/2 mated CBA/J mice and BABL/C mated CBA/J mice at different embryonic days. **(A)** Representative immunostaining images of FIB in decidua and labyrinth. **(B)** Relative FIB protein expression of decidua. **(C)** Relative FIB protein expression in labyrinth. **(D)** Representative immunostaining images of TF in decidua and labyrinth. **(E)** Relative TF protein expression of decidua. **(F)** Relative TF protein expression of labyrinth. Data were expressed as mean ± SD, *n* = 6 per group other than *n=*5 for E13.5-ENOX group. ^*^P < 0.05 and ^**^P < 0.01.

At E13.5, compared to normal group, the model group exhibited an 85.45% increase in decidual FIB expression (*P* < 0.01) and a 21.49% increase in labyrinth (*P* < 0.05). Similarly, TF protein expression was elevated by 56.23% in the decidua (*P* < 0.01) and by 23.21% in the labyrinth (*P* < 0.01). DYD down-regulated labyrinth TF expression by 22.96% compared to the model group (*P* < 0.01, **Figures 5E, F**).

### 3.6 The local fibrinolytic disorders in the placenta of DBA/2 mated CBA/J mice

In the decidua, collagen deposition increased 1.18-fold in the normal group from E9.5 to E11.5 (*P* < 0.05), then declined by 34.94% at E13.5 (*P* < 0.05 *vs*. E11.5). In contrast, model group showed progressive collagen accumulation in the decidua, with levels 1.05-fold higher than normal mice at E13.5 (**Figures 6A, C**).

In the labyrinth, collagen deposition remained unchanged in the normal group from E9.5 to E11.5. However, compared to the normal group, the model group showed a 66% increase at E9.5 and a 45.35% increase at E13.5 (both *P* < 0.05 , **Figures 6B, D**).

At E13.5, the model mice exhibited a 74.94% increase in placental PAI-1 level (*P* < 0.01 *vs*. normal group), accompanied by 105.17% and 45.35% increases in collagen deposition within decidua and labyrinths respectively (both *P* < 0.01 *vs*. normal group). DYD decreased the placental level of PAI-1 by 37.94% compared to the model mice (*P* < 0.05), whereas ENOX showed no effect on placental PAI-1 or t-PA levels (**Figures 6E, F**).

**Figure 6:**
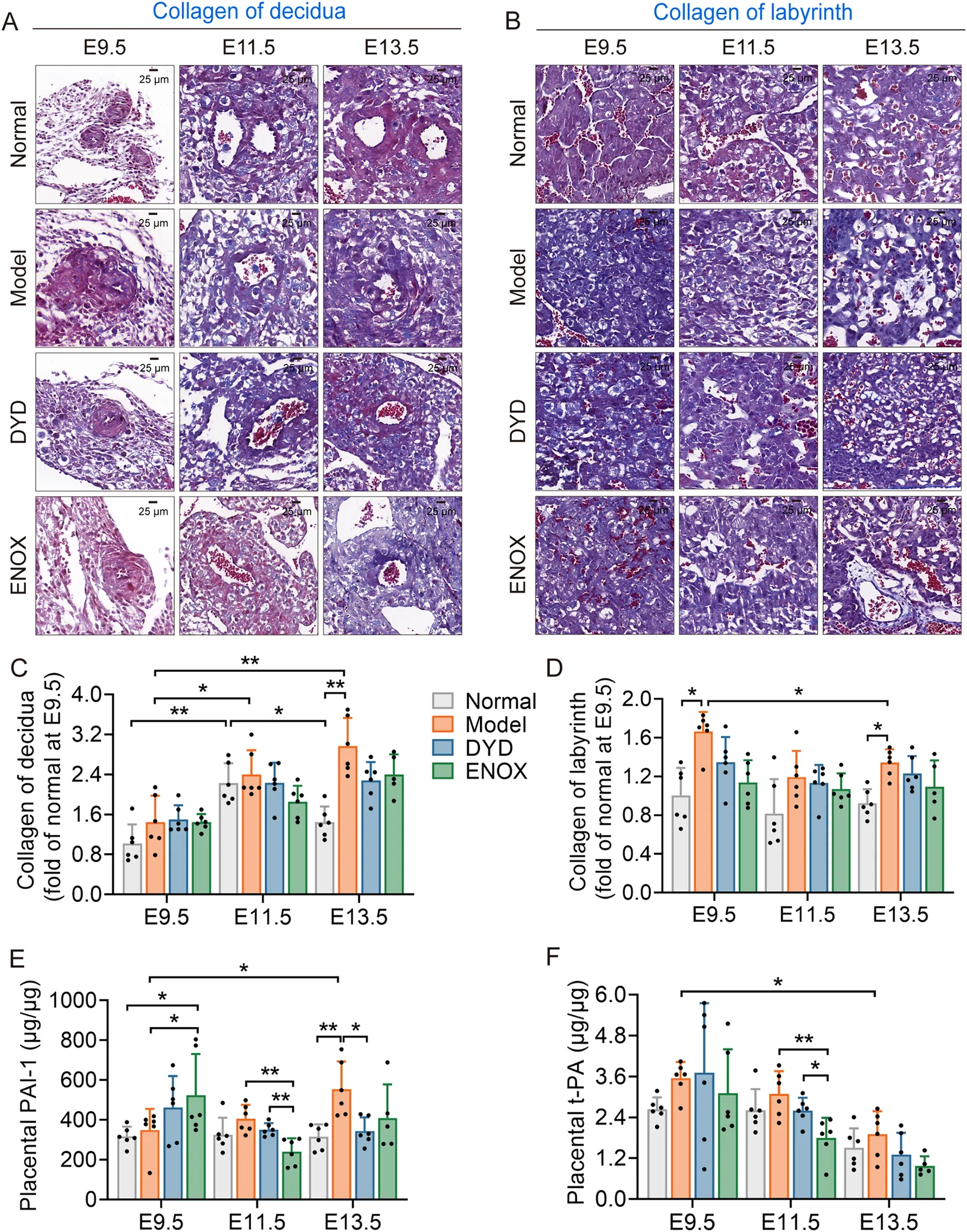
The changes on placental fibrinolytic function in DBA/2 mated CBA/J mice and BABL/C mated CBA/J mice at different embryonic days. **(A)** Representative images of collagen deposition in the decidua and labyrinths. **(B)** Representative images of collagen deposition in the decidua and labyrinths. **(C)** Relative level of collagen deposition in labyrinth. **(D)** PAI-1 level in the placenta. **(E)** The t-PA level in placenta. Data were expressed as mean ± SD, *n* = 6 per group other than *n=*5 for E13.5-ENOX group. ^*^P < 0.05 and ^**^P < 0.01.

### 3.7 The correlation of embryo resorption with placental congestion, resistance indices of the uterine and umbilical arteries, and coagulation-fibrinolysis parameters

Simple linear regression was used to assess the associations of embryo resorption rate with placental congestion, arterial resistance indices, and coagulation-fibrinolysis parameters. Placental congestion area was strongly positively correlated with embryo resorption rate (R^2^ = 0.7055, *P* < 0.0001, **Figure 7A**).

**Figure 7:**
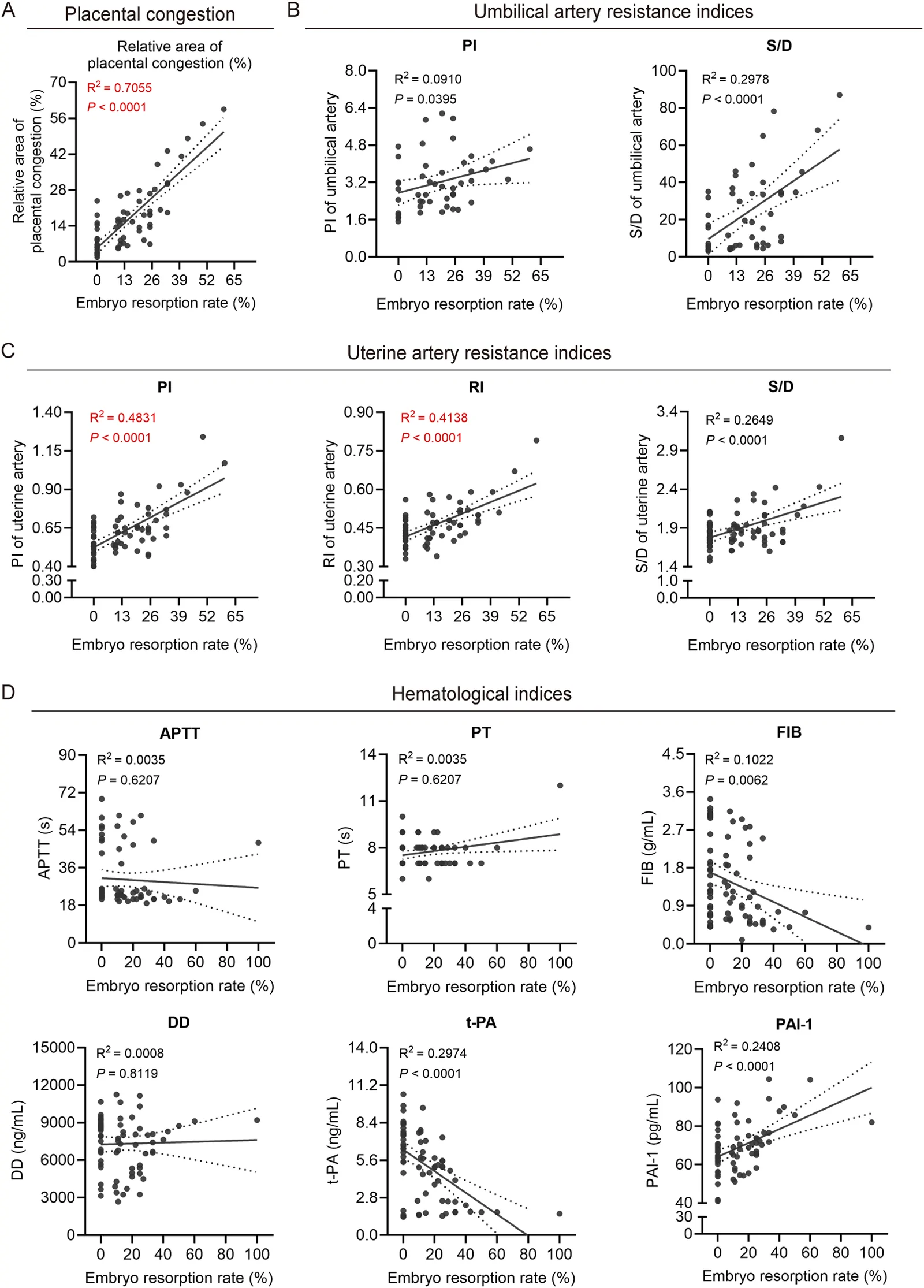
Correlation of embryo resorption with placental congestion, resistance indices of the uterine and umbilical arteries, and coagulation-fibrinolysis parameters. Linear regression analyses of embryo resorption rate against: **(A)** relative placental congestion area; **(B)** umbilical artery resistance indices (PI and S/D); **(C)** uterine artery resistance indices (PI, RI, and S/D); and **(D)** hematological parameters (APTT, PT, FIB, DD, t-PA, and PAI-1). Solid and dashed lines represent fitted regression lines and 95% confidence intervals, respectively. The coefficient of determination (R²) and corresponding *P* values are presented within each subplot.

Among umbilical artery parameters, the S/D ratio demonstrated a stronger positive correlation with embryo resorption rate (R^2^ = 0.2878, *P* < 0.0001), whereas PI showed only a weak positive correlation (R^2^ = 0.0910, *P* = 0.0395, **Figure 7B**). For the uterine artery, PI, RI, and S/D all exhibited significant positive correlations (R^2^ = 0.4831, 0.4138, and 0.2649, respectively, all *P* < 0.0001, **Figure 7C**).

Among coagulation-fibrinolysis parameters, APTT, PT, and DD showed no significant correlation with embryo resorption rate (**Figure 7D**), whereas FIB was negatively correlated (R^2^ = 0.1022, *P* = 0.0062). Both t-PA and PAI-1 demonstrated significant associations with embryo resorption rate, with t-PA showing a negative correlation (R^2^ = 0.2974, *P* < 0.0001) and PAI-1 a positive correlation (R^2^ = 0.2408, *P* < 0.0001).

### 3.8 The correlation of relative area of placental congestion with uterine/umbilical arterial hemodynamics and coagulation-fibrinolysis parameters

To elucidate the relationships among placental congestion, arterial resistance indices, and coagulation-fibrinolysis parameters, the linear regression analyses were performed. As shown in **Figure 8A**, both PI and S/D ratio were positively correlated with placental congestion area, with S/D showing a stronger correlation (R^2^ = 0.2796, *P* = 0.0001) than PI (R^2^ = 0.1248, *P* = 0.0149). Uterine artery PI, RI, and S/D ratio were all positively correlated with placental congestion area (**Figure 8B**, all *P* < 0.001), with PI showing the strongest association (R^2^ = 0.4275, *P* < 0.0001), followed by RI (R² = 0.3119, *P* < 0.0001) and S/D (R^2^ = 0.1629, *P* = 0.0005).

**Figure 8:**
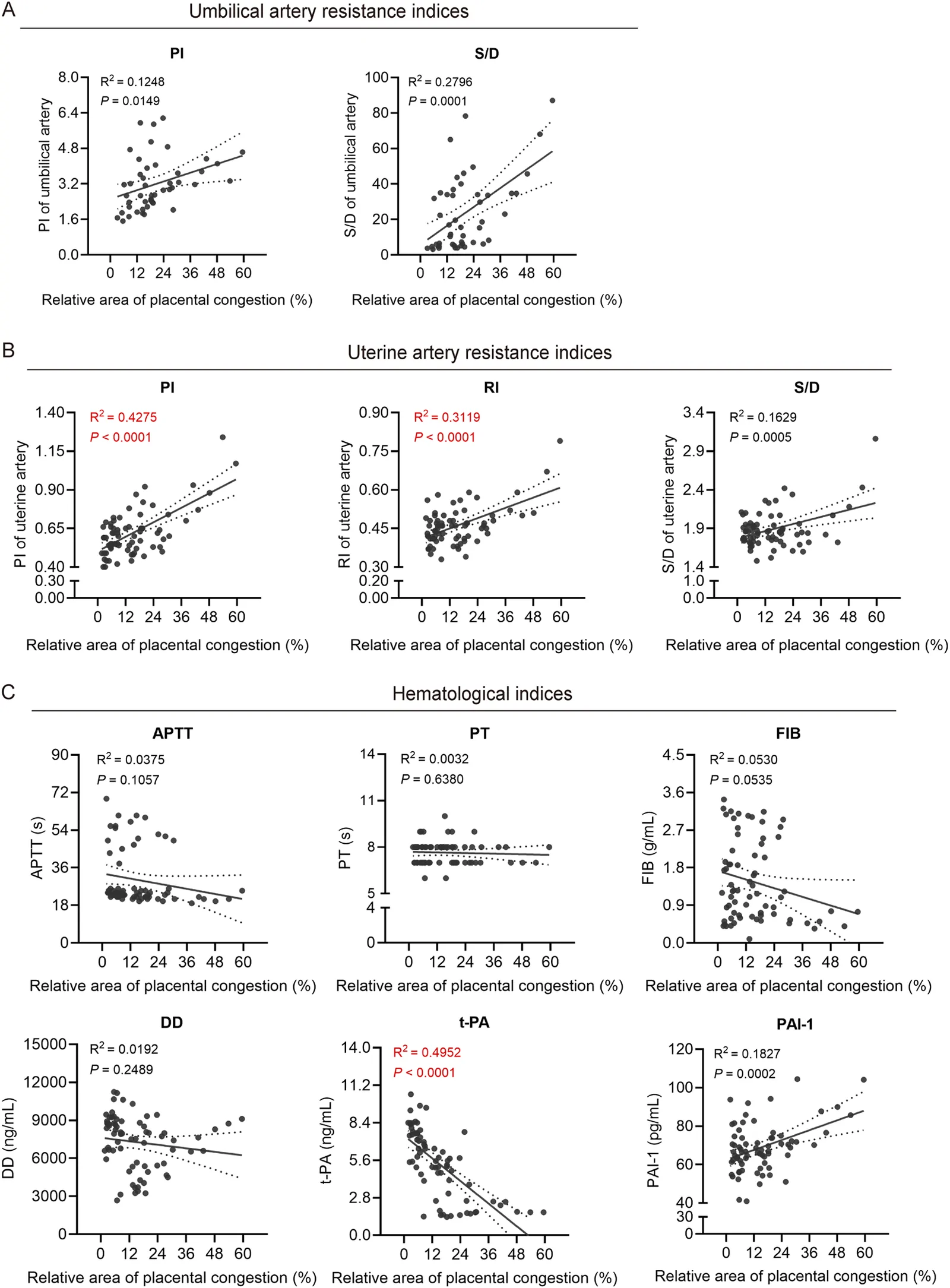
Correlation of the relative area of placental congestion with uterine/umbilical arterial hemodynamics and coagulation-fibrinolysis parameters. Linear regression analyses of the relative area of placental congestion: **(A)** umbilical artery resistance indices (PI and S/D); **(B)** uterine artery resistance indices (PI, RI, and S/D); and **(C)** hematological parameters (APTT, PT, FIB, DD, t-PA, and PAI-1). Solid and dashed lines represent fitted regression lines and 95% confidence intervals, respectively. The coefficient of determination (R²) and corresponding *P* values are presented within each subplot.

Among coagulation-fibrinolysis parameters (**Figure 8C**), APTT, PT, and DD showed no significant correlations with placental congestion area. Specifically, t-PA was negatively correlated (R^2^ = 0.4871, *P* < 0.0001), whereas PAI-1 was positively correlated (R^2^ = 0.2408, *P* < 0.0001). In contrast, FIB exhibited a borderline negative trend (R^2^ = 0.0530, *P* = 0.0535).

### 3.9 Hierarchical multiple linear regression analysis of embryo resorption rate

For embryonic resorption rate, an enter model was constructed incorporating uterine artery PI, RI, S/D, serum FIB, t-PA, and PAI-1. Among these, uterine artery PI (*β* = 0.300, *P* = 0.008), uterine artery RI (*β* = 0.258, *P* = 0.029), serum t-PA (*β* = −0.409, *P* < 0.001), and PAI-1 (*β* = 0.180, *P* = 0.023) were identified as independently associated factors (**Table 1**). Uterine artery S/D and serum FIB were not significantly associated with embryonic resorption rate in the multivariable model (*P* > 0.05). The final model explained 73.1% of the variance in embryonic resorption rate (Adjusted R^2^ = 0.705, *F*_(4,66)_ = 28.946, *P* < 0.001).

**Table 1:** Multivariate linear regression analysis of factors associated with embryonic resorption rate (*n* = 71).

Variables	Unstandardized B	SE	Standardized *β*	t	*P*-value	VIF
(Constant)	-27.141	8.811	—	-3.080	0.003	—
Uterine PI	27.993	10.148	0.300	2.758	0.008	2.816
Uterine RI	48.200	21.572	0.258	2.234	0.029	3.168
t-PA	-2.273	0.395	-0.409	-5.761	<0.001	1.196
PAI-1	0.200	0.086	0.180	2.330	0.023	1.418

R² = 0.731, Adjusted R² = 0.705, *F*_(4, 66)_ = 28.946, *P* < 0.001. All VIF values were below 5, indicating no severe multicollinearity. The Durbin–Watson statistic was 1.570, suggesting independence of residuals.

A separate enter model was constructed to evaluate factors of placental congestion area, including uterine artery PI, RI, S/D, serum t-PA, PAI-1, and APTT. In this model uterine artery PI (*β* = 0.327, *P* = 0.001), uterine artery RI (*β* = 0.238, *P* = 0.012), serum t-PA (*β* = -0.581, *P* < 0.001), and APTT (*β* = -0.182, *P* = 0.005) were identified as independently associated factors (**Table 2**). Uterine artery S/D and PAI-1 did not reach statistical significance in this model (*P* > 0.05). The model accounted for 80.0% of the variance in placental congestion area (Adjusted R²=0.778, F_(4,66)_=36.060, *P* < 0.001).

**Table 2:** Multivariate linear regression analysis of factors associated with placental congestion area (*n* = 71).

Variables	Unstandardized B	SE	Standardized *β*	t	*P*-value	VIF
(Constant)	-0.154	7.056	—	-0.022	0.983	—
t-PA	-2.904	0.308	-0.581	-9.434	<0.001	1.196
Uterine PI	27.385	7.904	0.327	3.465	0.001	2.552
Uterine RI	40.026	15.474	0.238	2.587	0.012	2.435
APTT	-0.172	0.06	-0.182	-2.889	0.005	1.248

R²= 0.800, Adjusted R^2^ = 0.778, *F*_(4, 66)_ = 36.060, *P* < 0.001. All VIF values were below 6, indicating no severe multicollinearity. The Durbin–Watson statistic was 1.562, suggesting independence of residuals.

Model diagnostics confirmed no violation of linear regression assumptions, with all VIF < 5, Durbin–Watson statistics of 1.570 and 1.562, and standardized residuals within ± 3 (Q-Q plots in **Supplementary Figure 3**). Hierarchical regression in 46 animals with complete umbilical data showed no significant improvement when adding umbilical artery PI and S/D (ΔR^2^ = 0.012 and 0.013, *P* = 0.409 and 0.618, respectively; **Supplementary Table 2**).

## 4 Discussion

In this study, we found that DBA/2 mated CBA/J mice exhibited elevated arterial resistance, hypercoagulation, and fibrinolytic suppression, consistent with the hypercoagulable and hypofibrinolytic features observed in RPL patients. DYD reduced embryo resorption, accompanied by improved uterine hemodynamics and reduced placental PAI-1. In contrast, ENOX failed to prevent embryo resorption but reduced placental congestion. Multivariate analysis further identified uterine artery resistance (PI and RI) and fibrinolytic markers (t-PA and PAI-1) as independently associated factors of embryo resorption and placental congestion (**Figure 9**).

**Figure 9:**
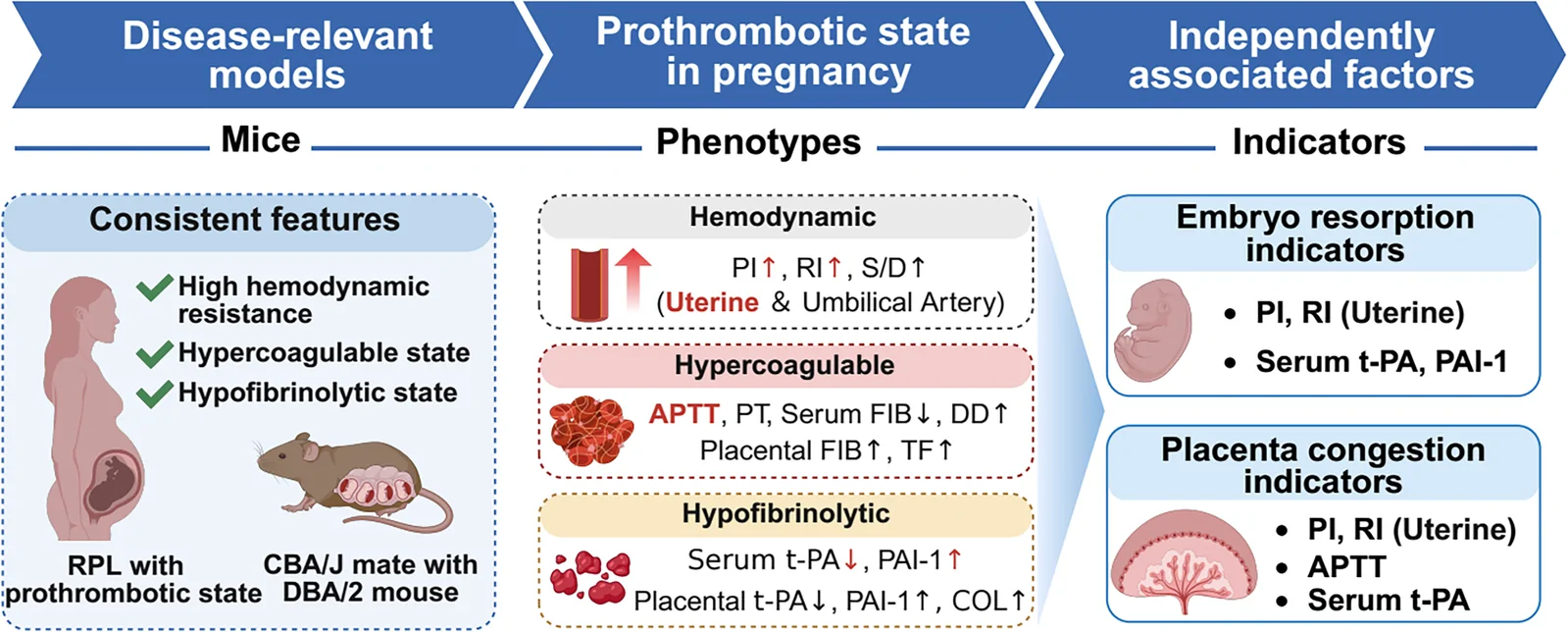
A concise summary of the research. The figure was created with BioRender.com (Agreement No.YG2A5MG4KR).

Among these findings, the strong positive correlation was observed between placental congestion area and embryo resorption rate is noteworthy. Histopathologically, the placental congestion corresponded to extensive erythrocyte accumulation in decidual sinuses and spiral arteries, accompanied by dense fibrin thrombi. These abnormalities may contribute to placental hypoperfusion and hypoxia, which are known to impair fetal development and promote resorption (Xie et al., 2025). Correlation does not imply causation, and congestion may share upstream triggers such as thrombosis. Nevertheless, the progressive worsening of these pathological changes from E9.5 to E13.5, together with the association between placental thrombosis and RPL in humans (Rodger et al., 2014), supports a contributory role of placental congestion in embryo loss.

In this study, uterine artery PI and RI increased progressively from E9.5 to E13.5 in model mice, paralleling the rise in embryo resorption rate. Both indices were independently associated factors of embryo resorption and placental congestion in the multivariate model. Uterine artery S/D was excluded due to collinearity with PI and RI. S/D is a ratio of systolic to diastolic velocity, whereas PI and RI integrate the full waveform and are more sensitive to changes in downstream vascular resistance (Li et al., 2024), which likely accounts for this collinearity.

Although umbilical artery PI and S/D showed significant univariate correlations with embryo resorption, they were excluded from the multivariate model due to poor model fit (embryo resorption: ΔR² = 0.012, *P* = 0.409; placental congestion: ΔR^2^ = 0.013, *P* = 0.618), and strong collinearity with uterine artery indices (r = 0.58-0.72, **Supplementary Figure S2**). The uterine arteries supply the uteroplacental circulation, and the umbilical arteries reflect fetal-side impedance and change secondarily when maternal perfusion is reduced (Bhide et al., 2021). Notably, elevations in uterine artery PI and RI have also been observed in RPL patients (Zhong et al., 2024). Together, these findings support uterine artery PI and RI as the primary hemodynamic factors in this model.

Beyond hemodynamic alterations, our findings revealed decreased t-PA and elevated PAI-1 in the circulatory system of model mice. Multivariable analysis showed that t-PA was the strongest negative associated factors of embryo resorption (*β* = -0.409) and placental congestion (*β* = -0.581), whereas PAI-1 independently associated embryo resorption (*β* = 0.180) but not congestion area. The reason may be that t-PA protects by dissolving fibrin and clearing thrombi, whereas PAI-1 promotes embryo loss primarily through non-coagulation pathways (Bardan et al., 2024). For example, PAI-1 may exert its effects on fibrin deposition and extracellular matrix remodeling at the maternal-fetal interface, consistent with the parallel rise in placental PAI-1 and collagen deposition observed in this study (Zhai et al., 2022). These findings are consistent with the observations of elevated PAI-1 and decreased t-PA in URPL patients (Bardan et al., 2024; Maghsudlu et al., 2024). Collectively, t-PA and PAI-1 emerged as the primary fibrinolytic factors of placental congestion and embryo resorption, respectively, in this model.

In the circulatory system, the model mice exhibited decreased serum FIB levels, along with increased placental FIB deposition and TF expression, as well as elevated serum DD levels. These findings reflect local coagulation activation with fibrin consumption at the maternal-fetal interface. In univariate analysis, serum FIB level showed a negative correlation with embryo resorption. However, this association was no longer significant after simultaneously including t-PA and PAI-1 as covariates in the multivariate regression model, while both t-PA and PAI-1 retained their independent significance. This pattern suggests that the circulating FIB level likely reflects the consumptive nature of local coagulation in the placenta rather than representing a primary causative determinant of embryo resorption, given that t-PA and PAI-1 function upstream of FIB in the fibrinolytic cascade, with FIB serving as their downstream substrate (Scarlatescu et al., 2025).

Additionally, APTT and PT showed no significant differences between the normal and model groups, consistent with clinical observations in RPL patients (Maher et al., 2025). Although APTT was associated with placental congestion in the multivariable model, its standardized regression coefficient was considerably smaller than that of uterine artery hemodynamic parameters, suggesting limited explanatory power for congestion. This association was largely attributable to data variability introduced by ENOX-induced APTT prolongation, reflecting the statistical manifestation of the anticoagulant effect of ENOX rather than an inherent link between the intrinsic coagulation pathway and placental congestion in the RPL pathological state.

In this study, DYD and ENOX were used to explore their respective effects in this miscarriage model. These two agents have distinct mechanisms, through progestogen-mediated pathways and anticoagulation, respectively, and were not intended for direct efficacy comparison. Notably, the differential effects of DYD and ENOX can be explained by the independently associated factors identified in this study. ENOX prolonged APTT and reduced placental congestion but failed to affect the factors validated by multivariate analysis. This result suggests that coagulation abnormalities or placental congestion alone are insufficient to account for embryo resorption in this model.

DYD improved uterine artery PI and RI, and reduced PAI-1. This reduction in PAI-1 correlates with DYD, but cannot be specifically attributed to fibrinolytic pathway regulation without additional mechanistic evidence. Nevertheless, DYD may exert these effects through progesterone-dependent regulation of uterine nature kill cells and their modulation on spiral artery remodeling (Alecsandru et al., 2021; Cuadrado-Torroglosa et al., 2024).

This study is based on a mouse model that recapitulates key pathological features of RPL. However, the clinical relevance of these four indicators (uterine artery PI and RI, as well as t-PA and PAI-1) remains to be validated in RPL patients exhibiting a prothrombotic state. Furthermore, it should be noted that term-pregnancy data (e.g., live-birth rate, litter size, neonatal weight, and postnatal survival) were not monitored in our study, and future investigations should address these parameters.

## 5 Conclusion

In conclusion, the DBA/2 mated CBA/J mouse model exhibits prothrombotic features characterized by fibrinolytic suppression and increased uterine artery resistance. Uterine artery PI and RI, serum t-PA and PAI-1 were identified as independently associated factors of embryo resorption and placental congestion. This model may serve as a useful tool for studying RPL with a prothrombotic state.

## Data Availability

The raw data supporting the conclusions of this article will be made available by the authors, without undue reservation.
