# Supplementary material for: Fibrinolytic suppression and increased uterine artery resistance are associated with embryo resorption in DBA/2 mated CBA/J mice
Source: Front Physiol. 2026 Oct 12;17:1942168. doi: 10.3389/fphys.2026.1942168 (PMC13696319; doi:10.3389/fphys.2026.1942168)
Supplement: Supplementary file 1 [file Table1.docx]

**Supplementary Table S1. Hierarchical regression analysis with and without umbilical artery Doppler parameters (*n* = 46)**

| Outcome | Model | Variables included: | R² | Adjusted R² | ΔR² | P for ΔR² |
| --- | --- | --- | --- | --- | --- | --- |
| Embryonic resorption rate | 1 | Uterine PI + RI + t-PA + PAI-1 | 0.736 | 0.711 | — | — |
|  | 2 | + Umbilical PI + S/D | 0.747 | 0.710 | 0.012 | 0.409 |
| Placental congestion area | 1 | Uterine PI + RI + t-PA + APTT | 0.788 | 0.75 | — | — |
|  | 2 | + Umbilical PI + S/D | 0.794 | 0.743 | 0.013 | 0.618 |
